# Interannual variability in early life phenology is driven by climate and oceanic processes in two NE Atlantic flatfishes

**DOI:** 10.1038/s41598-023-30384-7

**Published:** 2023-03-11

**Authors:** Ana Vaz, Ana Lígia Primo, Daniel Crespo, Miguel Pardal, Filipe Martinho

**Affiliations:** 1 Centre for Functional Ecology - Science for People & the Planet (CFE), TERRA Associate Laboratory, Department of Life Sciences, University of Coimbra, Calçada Martim de Freitas, 3000-456 Coimbra, Portugal; 2 Interdisciplinary Centre of Marine and Environmental Research, University of Porto, Terminal de Cruzeiros do Porto de Leixões, Avenida General Norton de Matos S/N, 4450-208 Matosinhos, Portugal

**Keywords:** Ichthyology, Biooceanography, Marine biology

## Abstract

Early life phenology is a crucial factor for population dynamics in a climate change scenario. As such, understanding how the early life cycle of marine fishes is influenced by key oceanic and climate drivers is of chief importance for sustainable fisheries. This study documents interannual changes in early life phenology of two commercial flatfishes: European flounder (*Platichthys flesus*) and common sole (*Solea solea*) from 2010 to 2015 based on otolith microstructure. Using GAMs, we looked for correlations of the North Atlantic Oscillation (NAO), Eastern Atlantic pattern (EA), sea surface temperature (SST), chlorophyl a concentration (Chla) and upwelling (Ui) variation with the onset of hatch, metamorphosis, and benthic settlement day. We concluded that higher SST, more intensive upwelling, and EA were coincident with a later the onset of each stage, while increasing NAO induces an earlier onset of each stage. Although similar to *S. solea*, *P. flesus* showed a more complex interaction with the environmental drivers, most possibly because it is at its southern limit of its distribution. Our results highlight the complexity of the relationship between climate conditions and fish early life history, particularly those with complex life cycles that include migrations between coastal areas and estuaries.

## Introduction

Climate change is significantly affecting the distribution, population dynamics and phenology of marine fishes at a global scale^1,2^, with contrasting effects throughout their life cycle^3,4^. Indeed, fish larvae are in general more sensitive to environmental variability, being more susceptible to climate change^5^. Also, heavily exploited fish populations by fisheries have been recognized as less resilient to climate change^6–8^, particularly those with complex life cycles such as flatfishes, which usually includes temporal and spatial segregation among life stages^9,10^. Flatfish juveniles often use coastal areas and estuaries as nursery grounds^11–14^, which they reach after migration from offshore spawning grounds. These migrations are coincident with the transition from pelagic to benthic life, which occurs through metamorphosis. This represents a critical phase in flatfish life cycle that ought to take place as fast as possible, as long as suitable environmental conditions are met^10,15^. Connectivity between these habitats plays a key role in recruitment^16,17^, which is mainly regulated by varying oceanic conditions that drive survival, growth, life history, food abundance and transport^18^.

As ectotherms, fish are directly exposed to oceanic processes, which are known key drivers in life history traits like growth, metabolic activity, and phenology. Indeed, fish move in response to abiotic (e.g. temperature, salinity, light and turbulence) and biotic factors (variations in abundance of food and predators)^19^. For instance, large-scale climate drivers such as the North Atlantic Oscillation index (NAO) and the East Atlantic pattern (EA) have been described as major long-term ecological drivers of fish abundance and growth^20,21^. The NAO is described as the main pattern of atmospheric variability in the Euro Atlantic, with its spatial pattern characterized by a meridional dipole in the pressure field between Azores and Iceland. Its positive phase is characterized by warm and wet conditions and in negative phase, the opposite pattern is registered^22^. In turn, the EA, also a dipole in the pressure field, when compared with NAO, has its centers of variability shift southern wards and more zonally oriented, and is also related to environmental variability in southern Europe^20^. On the other hand, seawater temperature modulates fish metabolic activity and thus, early life development processes like spawning, hatching, metamorphosis and settlement^14,23,24^. Additional extrinsic constraints for fish early life stages include oceanic phenomena like the wind-driven rising of nutrient-rich colder waters (i.e., upwelling^25^) and food availability^26^.

Changes in the onset and duration of fish early life history events such as hatching, metamorphosis and benthic settlement, have been reported at various temporal and spatial scales^12,14,27,28^. These studies were based on extracting temporally resolved information from sagittae otoliths (ear stones), whose microstructural landmarks allow for a precise estimation of early life history traits with a daily resolution^12,14^. However, the relationship between interannual variability in early life history and environmental variability has received far less attention^10,28,29^, which nonetheless provides a long-term link between fish early life history and climate variations.

This study uses two flatfishes as model species—the European flounder (*Platichthys flesus*; Linnaeus 1758) and the common sole (*Solea solea*, Linnaeus 1758), which have a broad geographical distribution in the North Atlantic: from the White Sea to the Mediterranean and Black Sea (72°N–40°N) for flounder^12,30^, and from the North Sea to Senegal (67°N–17°N) for sole^14^. These species share a life cycle typical of flatfishes that includes offshore batch spawning, after which pelagic larvae develop and metamorphose into benthic juveniles. During this period, they are transported towards coastal areas by favorable currents and wind-driven circulation^31,32^, and settle near estuaries and shallow coastal areas, where juveniles usually concentrate. Despite being models for several studies involving larval transport, juvenile recruitment and connectivity^12,14,33,34^ there is a significant lack of knowledge on the interannual variability in early life history when facing changing oceanic and atmospheric processes, which will necessarily better inform management and protection measures. These species were chosen given their reported shifts in geographical distribution towards higher latitudes in response to the warming of the sea over the last decades^11,35^.

The aim of this study was to investigate the influence of climate and oceanic variability on early life history events of two highly commercially valued flatfishes (European flounder and common sole) on the Portuguese Atlantic coast over a 6-year period (2010–2015), to test the hypothesis that variability in extrinsic forcing resulted in changes in fish hatching, metamorphosis and settlement phenology.

## Material and methods

### Study area and fish sampling

**Figure 1:**
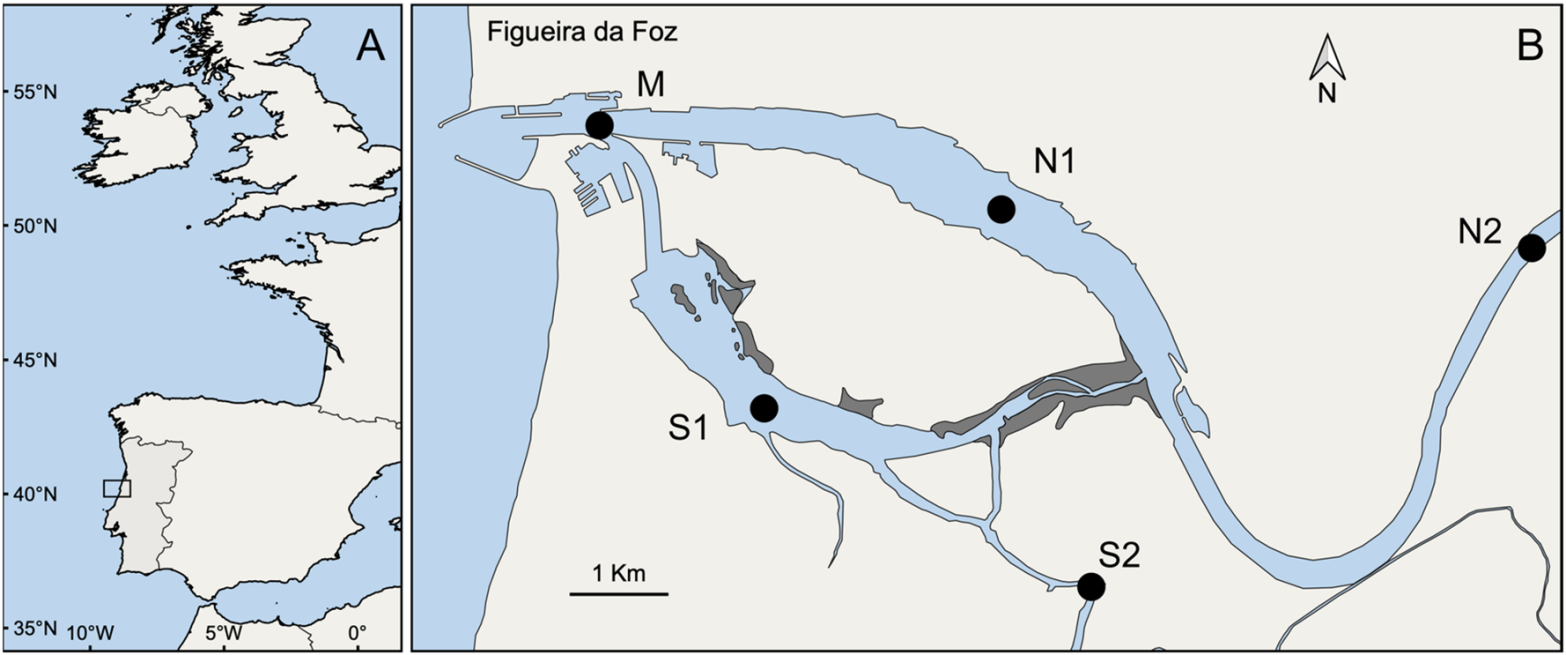
Geographical location of the Portuguese Atlantic coast (**A**) and the sampling stations in the Mondego estuary (**B**), denoted by the letters M, N1, N2, S1 and S2. Maps were created using QGIS 3.10.10 software (https://qgis.org).

The Portuguese coast is located on the Iberian Peninsula and is the northern limit of the Canary Current Upwelling System (Fig. 1A). This area is characterized by seasonal variability in currents and water temperature, which include a summer wind-driven upwelling-type shelf circulation with associated equatorward surface currents^36^, a winter poleward surface circulation designated as Iberian Poleward Current (IPC) and a surface water mass fed by river runoff (Western Iberia Buoyant Plume; WIBP)^25,37^. Such highly dynamic oceanic conditions are known to influence the connectivity and survival of fish early life stages^25,29^.

Juvenile 0-group *P. flesus* and *S. solea* were obtained in the Mondego estuary, located in the western Atlantic coast of Portugal (40º 08’ N, 8º 50’ W) (Fig. 1B). This temperate estuary has an area of 8.6 Km^2^ with nearly 75% of intertidal mudflats, and is divided in two hydrologically distinct arms (northern and southern) in its terminal part, which join again near the mouth^38^. Previous work has established the estuary as a key nursery area for this and other marine fish species^13,14,29,32,38,39^. Sampling occurred during spring/summer from 2010 to 2015 in 5 stations (Fig. 1) to ensure that the whole estuary was represented. Fish samples were obtained at night with a 2-m beam trawl equipped with one tickler chain and 5 mm mesh size in the cod end. At each station, 3 hauls were towed at a speed of 2 knots, covering a minimum area of 500 m^2^, and the fish caught were immediately transferred to iceboxes.

Once in the laboratory, we registered fish total length (TL; mm) and wet weight (WW, g), and removed the respective sagittae otoliths, cleaned and stored them in eppendorfs until further analysis. This study was carried out following the recommendations of Directive 2010/63/EU, and fish handling protocols were approved by the Portuguese National Authority for Animal Health (DGAV; Ref 0421/000/000/2017). No live animals were used in this study, and both species are neither protected nor endangered. Reporting in the manuscript follows the recommendations in the ARRIVE guidelines^40^.

**Figure 2:**
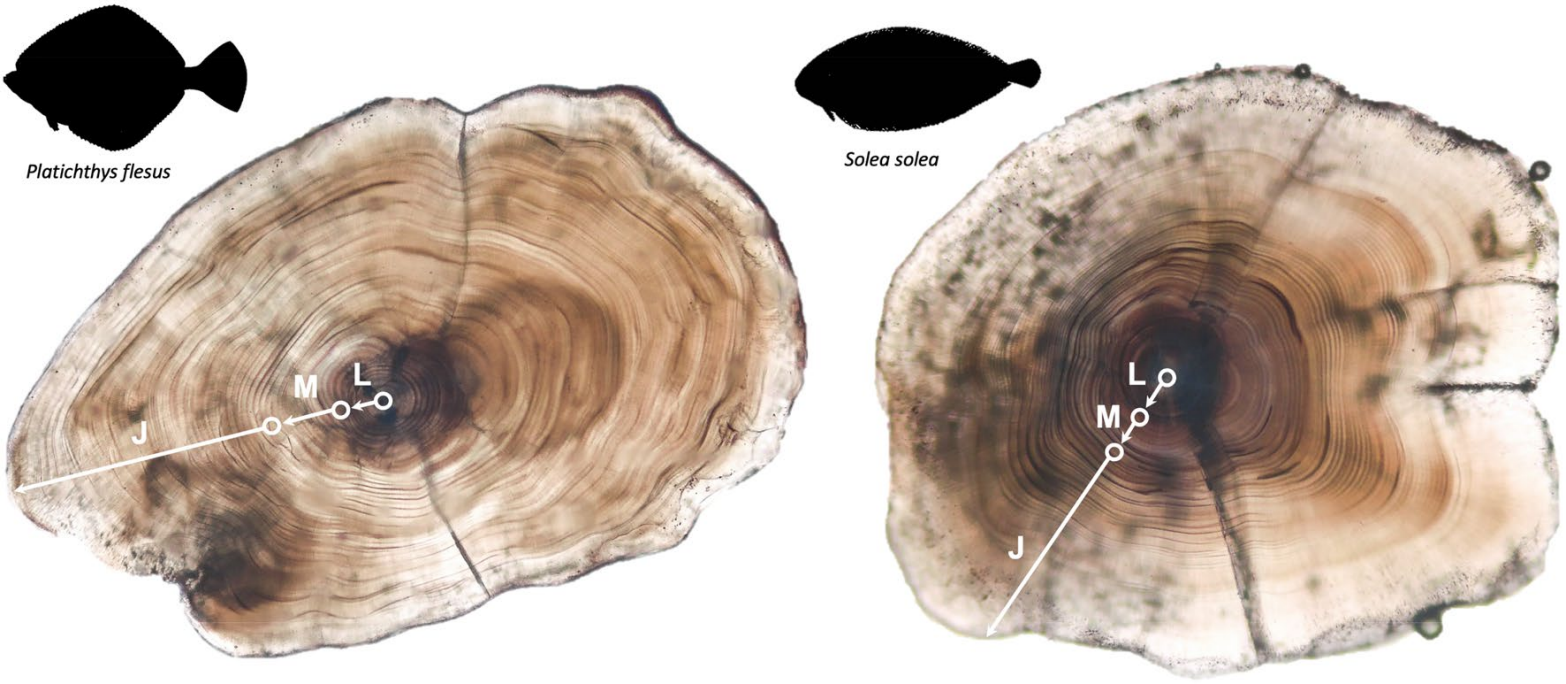
0-group European flounder (*Platichthys flesus*) and common sole (*Solea solea*) sagittae otoliths at 50× magnifications, highlighting the daily growth increments and the early ontogenic development stages: L—larval (pelagic), M—metamorphosis, J—juvenile (benthic). Circles indicate the respective onset day for each stage.

From the selected specimens, otoliths were mounted sulcus up on microscope slides with Crystalbond 509 and polished on the sagittal plane using P4000 (5 µm) Buehler silicon carbide grinding paper until the daily rings were clearly visible from the core to the edge. All daily increment counts were made using a light microscope at 100× and 400× magnifications for the peripheral areas, and at 1000× magnifications for the core. Otolith microstructure analysis was used to determine fish age, the main early life history events: hatching, metamorphosis and benthic settlement day, and the duration of the larval and metamorphosis stages, using the back-calculation method^12,14,27^ (Fig. 2). Briefly, the pelagic larval stage (L) corresponds to the otolith core and the metamorphosis stage to the daily rings between the innermost and outermost accessory growth centers (accessory primordia) (M). The benthic juvenile stage (J) corresponds to the number of daily rings between the first uninterrupted ring after the outermost accessory primordia and the otolith edge. Hatch day was determined as the first ring in the otolith core, day at metamorphosis the first day when accessory primordia are accounted for, and settlement day the first ring of the juvenile benthic stage (Fig. 2).

### Environmental data

For the 6-year study period (2010–2015), a set of environmental variables, were collected with different temporal resolutions and spatial scale of operation: Sea surface temperature (SST; °C), Chlorophyll a (Chl a; mg L^−1^), North Atlantic index (NAO), East Atlantic pattern (EA) and Upwelling index (Ui; m^3^ s^−1^ km^−1^). These variables were selected based on their role as key drivers of marine life history traits like growth, metabolic activity and phenology^25,29^. SST and Chl a data consisted of 8 day means obtained within a radius of 20 km near the Mondego estuary. Data was obtained from US NASA Oceancolor Web (https://oceancolor.gsfc.nasa.gov). NAO and EA data consisted of monthly means and were acquired from the US NOAA National Weather Service Climate Prediction Centre (https://www.cpc.ncep.noaa.gov). Lastly, monthly means of the Upwelling index were obtained for the Figueira da Foz FNMOC station from the Spanish Oceanographic Institute (IOE; http://www.indicedeafloramiento.ieo.es).

### Data analysis

A total of 219 flounder and 204 sole 0-group juveniles were used in this study (Table 1). Previous work had determined the linear relationship between fish total length and otolith length in both species^28,41^. Hence, a linear regression model between total length (TL) and the estimated Age (days) in both species was performed, considering the year of capture (Year) as a fixed factor, and including the interaction between Age and Year to investigate possible year-dependent growth patterns. Then, Kruskal–Wallis ANOVAs followed by pairwise Wilcoxon tests with a Bonferroni correction were performed to investigate interannual differences in hatch, metamorphosis, and settlement dates for both species.

**Table 1:** Summary of the number of *Platichthys flesus* and *Solea solea* specimens per year (n), with the respective range in total length (TL, cm), wet weight (WW, g) and age (days).

Year	*Platichthys flesus*				*Solea solea*			
	n	TL	WW	Age	n	TL	WW	Age
2010	10	4.7–7.5	0.8–4.2	86–107	29	5.0–9.8	0.7–5.3	66–120
2011	34	5.1–9.3	0.8–8.3	88–146	34	2.8–10.2	0.2–7.9	68–89
2012	49	4.9–9.9	0.8–7.5	88–153	44	5.0–9.9	0.3–4.7	61–94
2013	50	1.9–9.1	0.3–7.1	49–142	32	2.5–7.7	0.1–8.3	45–87
2014	28	3.0–9.8	0.6–9.1	70–150	26	3.7–9.5	0.3–6.3	54–82
2015	47	3.1–8.1	0.9–2.6	69–126	39	3.2–9.8	0.83–5.2	51–87

The relationship between the onset of each stage and the environmental factors (SST, Chla, NAO, EA and Ui) was investigated with generalized additive models (GAM; Gaussian family, Identity link function), as preliminary analyses determined a non-linear relationship between the dependent and independent variables^38,42^. Chla data was log-transformed to reduce the skewness of the original data. Prior to further analyses, the collinearity between the environmental predictors was assessed with a Pearson correlation coefficient. The only case when two variables had a correlation coefficient above 0.4 occurred in flounder hatch day—NAO and Ui, which led us to not include the latter. All environmental variables were included in the subsequent analyses. Considering the different temporal resolutions of the environmental data, all hatch, metamorphosis, and settlement dates were assigned to the corresponding 8-day (SST, Chla) and monthly (NAO, EA, Ui) values.

GAM development included testing the correlation between all possible combinations of the environmental factors (NAO, EA, Ui, SST, Chla) and year of capture on the early life history of flounder and sole, and the models with lower AIC were selected. These analyses were performed in R software (R development Core Team, 2018) using the *mgcv* package^43^, considering a 5% significance level.

## Results

### Environmental conditions

**Figure 3:**
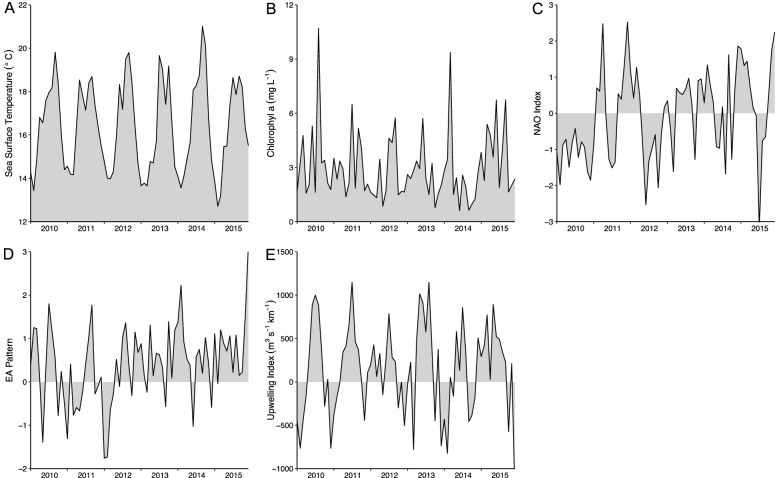
Monthly values for the selected environmental factors in the Portuguese Atlantic coast (2010–2015): (**A**) Sea Surface Temperature (SST; °C); (**B**) Chlorophyll a (Chla; mg L^−1^); (**C**) North Atlantic Oscillation index (NAO); (**D**) East Atlantic Pattern (EA); (**E**) Upwelling index (Ui; m^3^ s^−1^ km^−1^).

From 2010 to 2015, the annual mean of SST was higher in 2014 (16.70 °C) and lower in 2013 (15.90 °C) (Fig. 3A). For Chlorophyll a, the highest value was registered in 2015 (3.79 mg m^−3^) and the lowest in 2012 (2.52 mg m^−3^) (Fig. 3B). Coastal upwelling ranged between 198.4 m^3^ s^−1^ km^−1^ (2015) and the particularly low values in 2010 and 2014 (50.3 m^3^ s^−1^ km^−1^ and 2.8 m^3^ s^−1^ km^−1^, respectively; Fig. 3C). Both the NAO and EA showed a general increasing trend in mean annual values towards the end of our study period (Fig. 3D, E). The mean NAO index varied between 0.43 in 2015 and − 1.15 in 2010 (Fig. 3D), while EA ranged between 0.57 in 2014 − 0.02 in 2011 (Fig. 3E).

### Otolith microstructure analysis

**Figure 4:**
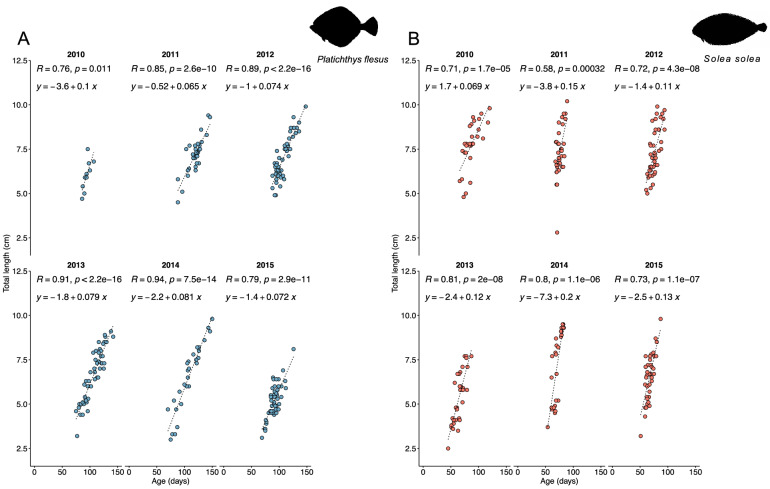
Linear regression between total length (cm) and age (days) for 0-group juvenile *P. flesus* (**A**) and *S. solea* (**B**) between 2010 and 2015.

The linear regression between total length and age showed that for *P. flesus*, both age and year of capture were significant (*p* = 2.2e^−16^ and *p* = 1.447e^−06^, respectively). However, their interaction was not significant (*p* = 0.3481), indicating that the slopes were similar between years (Fig. 4A). In *S. solea*, a significant interaction for age and year was found: 2010 was different from all years, 2011 and 2012 were different from 2015 (2015p = 2.2e−16, *p* = 2.395e−06 and *p* = 0.0003305, respectively) (Fig. 4B).

### Early life history

**Figure 5:**
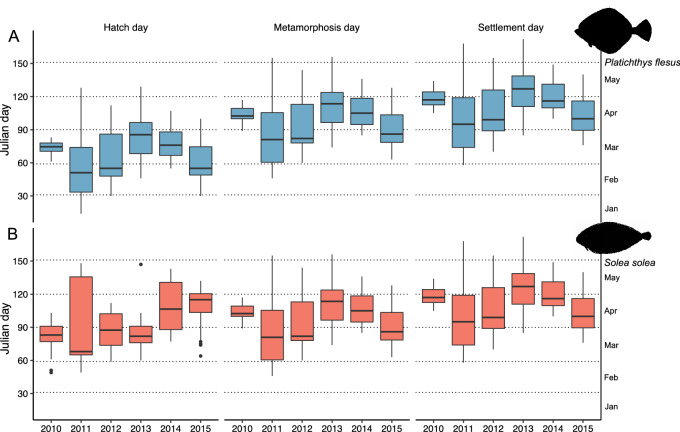
Hatch, metamorphosis, and settlement day distribution boxplots for (**A**) *Platichthys flesus and* (**B**) *Solea solea* from 2010 to 2015 (in Julian days). The secondary Y-axis shows the corresponding month. The boxplot horizontal thick lines represent the median value and each box the interquartile range from 25 to 75%. The bars represent the largest value within 1.5 times the interquartile range above the 75th percentile and the smallest below the 25th percentile respectively, and the outliers are represented by the black dots. The secondary Y-axis represent the respective month.

Flounder hatch onset day varied between years (H = 38.561; *p* = 2.91e^−07^) (Fig. 5). The earliest and latest hatch day were registered in 2011 (22nd January and 24th April). In more detail, hatch day distributions were significantly different between 2011 and 2013 (*p* = 0.0003), 2012 and 2103 (*p* = 0.001), 2011 and 2014 (*p* = 0.028), 2013 and 2015 (*p* = 5.7e^−05^), and 2014 and 2015 (*p* = 0.009) (Table S1). Significant differences were also found between years on the onset of metamorphosis in *P. flesus* (H = 36.864; *p* = 6.377e^−07^) (Fig. 5). In line with the previous stage, the onset period of metamorphosis was also shorter in 2010. The earliest and latest days at metamorphosis also occurred in 2011, at 28 of January and 10 of April, respectively. Finally, differences between years for the onset of settlement (H = 39.602; *p* = 1.796e^−07^) were also found (Fig. 5). The widest onset period of settlement was in 2011 (27 of February to 17 June), and the shortest in 2010 (15 of April to 14 of May). Significant differences between years for metamorphosis and settlement were also observed for the same years as in hatch day (Table S1).

*Solea solea* also presented different hatch day periods between years (H = 39.768; *p* = 1.663e^−07^) (Fig. 5), which was smaller in 2013, with a duration from the 1^st^ of March to the 27th of May, and longer in 2011, from the 18th of February to 29th of May. The onset of metamorphosis was also significantly different between years (H = 37.403; *p* = 4.972e^−07^): the onset period of this stage was longer in 2014 (11th of April to 27th of September) and shorter in 2012 (21^st^ of March to 20th of May). Finally, the day at settlement was also different between years (H = 38.359; *p* = 3.195e^−07^), with the cohort of 2011 presenting the widest period (16th of March to 4th of June), and the 2013 cohort the shortest one (9th of April to 27th of May). Significant differences were found for the three stages between 2014 and 2014, 2013 and 2013, and between 2015 and 2010, 2012 and 2013 (Table S2).

### Response to the main environmental drivers

The relationship between several environmental factors (SST, Chla, Ui, NAO and EA) was investigated for all *P. flesus* and *S. solea* early life stages along the period between 2010 and 2015, using GAMs.

**Figure 6:**
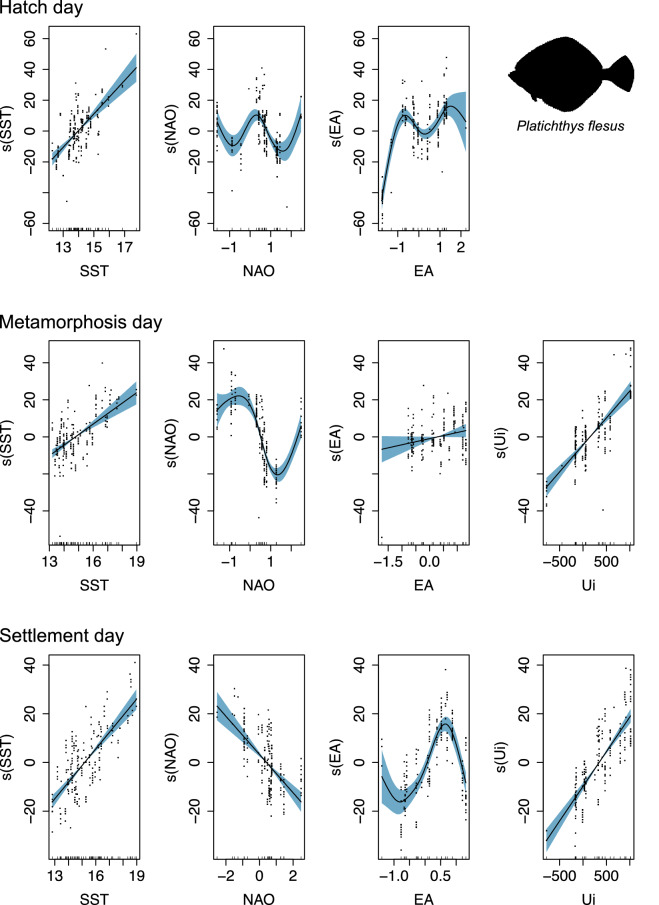
Generalized Additive Models (GAM) smoother response curves of the environmental drivers explaining the variation in *Platichthys flesus* hatch, metamorphosis, and settlement day for the period between 2010 and 2015. SST—Sea Surface Temperature (°C); Ui—Upwelling index (m^3^ s^−1^ km^−1^); NAO—North Atlantic Oscillation; EA—Eastern Atlantic Pattern. Black dots depict the partial residuals for each term. The uncertainty bands denote the 95% confidence intervals for each term.

For flounder, the hatch, metamorphosis and settlement dates increased when higher seawater temperatures, more intense upwelling events and positive EA occurred, leading to a later development (Fig. 6). In contrast, flounder hatch, metamorphosis and settlement phenology decreased when increasing NAO was registered. Adjusted R^2^ and deviance explained for the three models regarding each life stage were always superior to 0.77 and 78.10%, respectively (Table 2).

**Table 2:** Generalized additive model (GAM) outputs for parametric coefficients and smooth terms testing the interaction between environmental variables and *Platichthys flesus* hatch, metamorphosis, and settlement days between 2010 and 2015.

Life Stage	Parametric coefficients					Smooth terms						
	Year	Estimate	Std. Error	t value	Pr( >|t|)	Covariate	edf	Ref.df	F	*p* value	R^2^ (adj)	Deviance explained (%)
Hatch	2010	58.909	6.033	9.764	< 2E−16*	SST	1.000	1.000	89.430	< 2E−16*	0.77	78.10
	2011	− 8.749	6.918	− 1.265	2.07E−01	NAO	3.508	4.111	12.260	2.58E−09*		
	2012	22.076	7.982	2.766	6.20E−03*	EA	3.585	4.186	46.430	< 2E−16*		
	2013	11.375	5.280	2.154	3.24E−02*							
	2014	9.568	6.336	1.510	1.33E−01							
	2015	16.164	7.042	2.295	2.27E−02*							
Metamorphosis	2010	79.914	4.198	19.038	< 2E−16*	SST	1.000	1.000	52.915	5.90E−12*	0.84	84.60
	2011	4.367	4.418	0.988	3.24E−01	NAO	3.160	3.768	62.262	< 2E−16*		
	2012	28.931	4.432	6.527	5.72E−10*	EA	1.000	1.000	3.254	7.28E−02*		
	2013	25.535	5.510	4.634	6.57E−06*	Ui	1.000	1.000	107.682	< 2E−16*		
	2014	23.537	4.522	5.205	4.93E−07*							
	2015	15.638	4.823	3.242	1.40E−03*							
Settlement	2010	107.498	3.883	27.687	< 2E−16*	SST	1.000	1.000	155.010	< 2E−16*	0.85	85.80
	2011	10.303	4.261	2.418	1.65E−02*	NAO	1.000	1.000	63.760	5.34E−14*		
	2012	11.281	3.996	2.823	5.23E−03*	EA	3.209	3.897	32.410	< 2E−16*		
	2013	10.236	4.560	2.245	2.59E−02*	Ui	1.000	1.000	187.370	< 2E−16*		
	2014	− 1.581	4.333	− 0.365	7.16E−01							
	2015	− 8.700	5.133	− 1.695	9.16E−02*							

Significant terms are highlighted with *.

**Figure 7:**
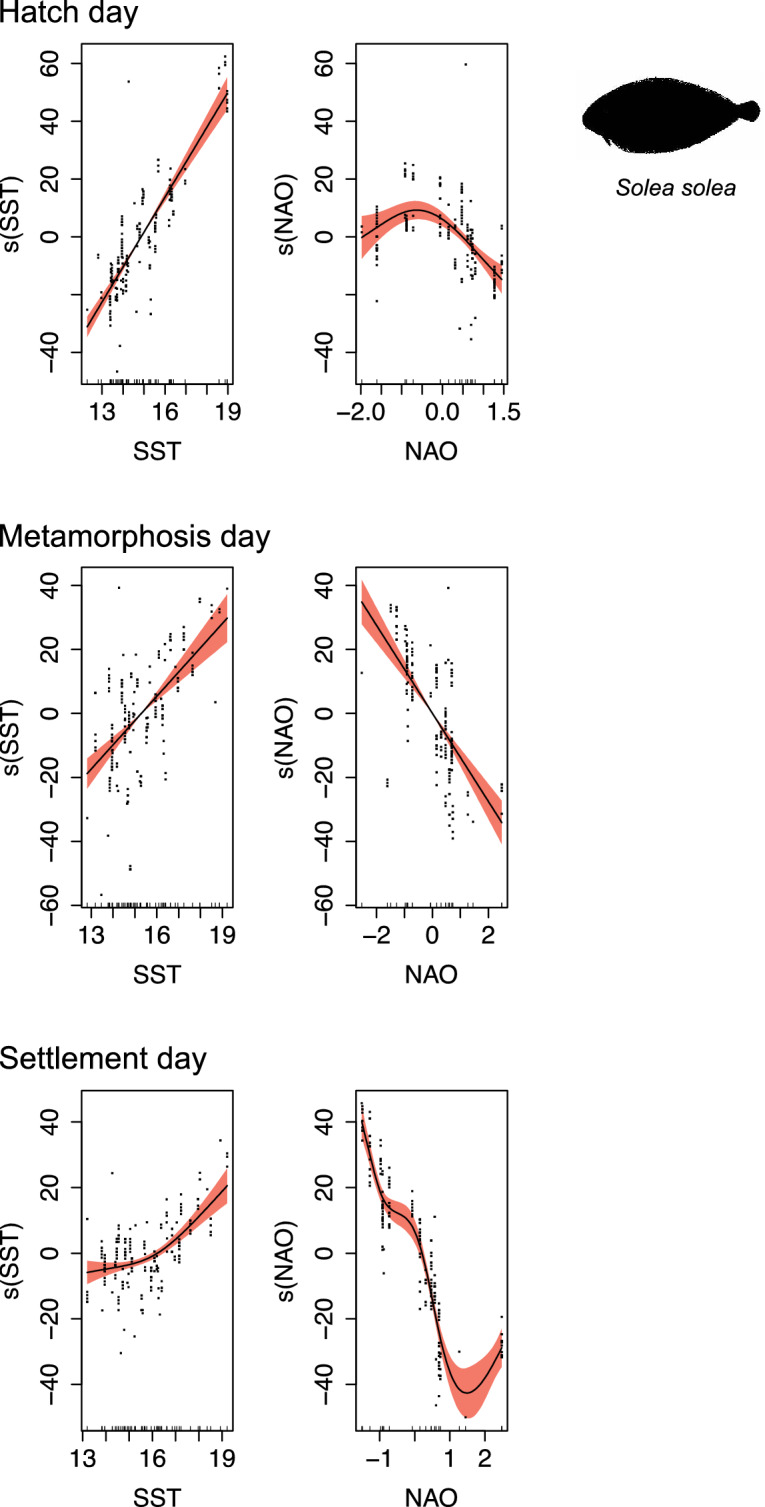
Generalized Additive Models (GAM) smoother response curves of the environmental drivers explaining the variation in *Solea solea* hatch, metamorphosis, and settlement day for the period between 2010 and 2015. SST—Sea Surface Temperature (°C); NAO—North Atlantic Oscillation. Black dots depict the partial residuals for each term. The uncertainty bands denote the 95% confidence intervals for each term.

In sole, there was also a consistent concordance among life stages of SST and the large-scale climate index NAO (Fig. 7). Like flounder, higher water temperatures were coincident with later hatch, metamorphosis and settlement, while increasing NAO index values were found with earlier life stages. Adjusted R^2^ and deviance explained for the three models regarding each life stage were always superior to 0.63 and 64.40%, respectively (Table 3).

**Table 3:** Generalized additive model (GAM) outputs for parametric coefficients and smooth terms testing the interaction between environmental variables and *Solea solea* hatch, metamorphosis, and settlement days between 2010 and 2015.

Life stage	Parametric coefficients					Smooth terms						
	Year	Estimate	Std. Error	t value	Pr( >|t|)	Covariate	edf	Ref.df	F	*p* value	R^2^ (adj)	Deviance explained (%)
Hatch	2010	70.354	2.465	28.543	< 2E−16*	SST	1.000	1.000	318.880	< 2E−16*	0.81	81.70
	2011	8.599	3.371	2.551	1.15E−02*	NAO	1.930	2.419	18.330	7.43E−09*		
	2012	34.296	3.476	9.866	< 2E−16*							
	2013	24.135	3.067	7.869	2.37E−13*							
	2014	37.394	3.105	12.044	< 2E−16*							
	2015	33.574	3.549	9.461	< 2E−16*							
Metamorphosis	2010	83.097	2.924	28.416	< 2E−16*	SST	1.000	1.000	63.710	6.53E−14*	0.63	64.40
	2011	33.462	3.781	8.850	5.46E−16*	NAO	1.000	1.000	100.970	< 2E−16*		
	2012	37.981	3.991	9.517	< 2E−16*							
	2013	32.446	4.041	8.028	9.29E−14*							
	2014	33.557	3.952	8.491	5.29E−15*							
	2015	52.541	3.802	13.820	< 2E−16*							
Settlement	2010	88.190	1.869	47.180	< 2E−16*	SST	1.613	2.017	32.110	3.79E−13*	0.88	88.80
	2011	37.184	2.889	12.870	< 2E−16*	NAO	3.922	4.581	171.690	< 2E−16*		
	2012	34.789	2.432	14.310	< 2E−16*							
	2013	52.199	3.031	17.220	< 2E−16*							
	2014	37.049	2.460	15.060	< 2E−16*							
	2015	56.298	2.742	20.530	< 2E−16*							

Significant terms are highlighted with *.

## Discussion

This study documented how key climate and oceanic factors may be linked to early life phenology of two commercially important flatfishes—*P. flesus* and *S. solea* in the Portuguese Atlantic coast. Although previous work addressed the onset of early life stages at larger spatial scales^12,14^, very few have attempted to understand how environmental drivers are correlated with the early life cycle of *S. solea* and *P. flesus*^10,44^. As such, this work provides a stepping stone for the comparison between two flatfishes with distinct biogeographical affinity (*P. flesus*—temperate; *S. solea*—sub-tropical), disentangling the relationship between climate and oceanic conditions on fish early life history, which coincides with their migration from the spawning grounds towards estuarine nurseries. Studies of this nature are important in the view of climate change, contributing to a better understanding of how species early life history and phenology are shaped by climate variability, as well as to predicting possible consequences to species recruitment^13,28,29^ and stock structure in the long term. Unravelling the impacts of climate variability on the early life history of marine fishes will also contribute to the development of more sustainable management and exploitation strategies^10^.

Our results showed that the early life cycle of *P. flesus* (i.e. hatching, metamorphosis, and settlement) suffered changes when different SST and upwelling conditions were recorded, as well as with the large climate drivers NAO and EA pattern. For *S. solea*, SST and the NAO where the only relevant environmental drivers across early life stages. Interestingly, SST and the NAO had a consistent relationship among species and throughout their early life history.

Water temperature plays a key role in the life cycle of marine fishes, being responsible for most metabolic processes involved in vital processes, somatic growth, movements, and survival^12,14^. For *P. flesus* our results showed that hatching occurred at sea surface temperatures between 13.9 °C (2^nd^ of March 2010) and 15.3 °C (14th of January 2011), and in *S. solea* between 14.6 and 15.3 °C (18th March 2014 and 18th February 2011, respectively). In both species, hatching occurred near winter’s end and early spring, which corroborates previous studies, corresponding to the reproductive period of these species^14^. In fact, it has been demonstrated that reproductive migrations and spawning on several species, including those in the current study, are influenced by water temperature^14,45,46^. A synchronous effect of SST on *P. flesus* and on *S. solea* early life history was observed, with a positive linear relationship between SST and the onset of each stage, indicating a correlation between warmer water temperatures and a later onset hatch, metamorphosis, and settlement days. Indeed, both species displayed inter-annual variability in hatch dates, and the later hatching in both species took place in 2011, which corresponded to the warmest water temperatures experienced (*P. flesus,* 17.8 °C; *S. solea*, 19.0 °C).

One of the key aspects of climate change on marine ectotherms concerns the effect of warming water temperatures on early life stages. Indeed, studies have demonstrated the potential of global warming to reduce population connectivity and dispersal in several marine fishes^4,23^, by reducing the pelagic larval duration (PLD) and increasing growth rates^24^. Such changes in development, as well starvation, may induce a lower swimming capacity^47^ which can end up affecting the dispersal and recruitment^48^. Our study provides additional insights on the expected impacts of global warming, which include the delaying of hatch phenology in these two commercially important species. Such temporal displacement might, for instance, lead to the decoupling of predator–prey interactions (i.e., flatfish larvae and zooplankton abundance^49,50^), as well as to higher energy expenditure to overcome the energetic costs of survival in a warmer ocean^51^. Still, the effect of ocean warming will vary according to the position within a species’ geographical distribution gradient, as populations show distinct phenotypical responses in relation to the latitudinal temperature cline. For instance, several authors showed that the timing and duration of early life history stages (as well as growth) of these species and those with similar life cycle vary with the prevailing temperature regimes^12,14,29,33^, showing a negative relationship between water temperature and hatch day across species geographical distribution ranges^12,14^.

There is a consensus that spawning and early life stages have the narrowest thermal window within a fish life cycle^52^. As such, relatively small variations in water temperature as those reported here are likely to contribute to a temporal shift in spawning, and consequently, in larval hatching, metamorphosis, and settlement. Previous work has indicated that earlier spawning in warmer waters occurs mainly through faster gonadal development in adults^53^. However, our results point to the opposite direction, in which warmer waters were coincident with latter hatching in flounder and sole, which can be related to (but not exclusively) reduced fertility and developmental success, egg and spermatozoa morphology and composition^54^. Still, fish spawning and hatching can also be modulated by the timing and duration of exposure to unexpected water temperatures in a fish life cycle^54^, as well as other important factors such as day length during pre- and spawning stages^55^. A similar relationship with water temperature was recently demonstrated for the European seabass *Dicentrarchus labrax* in the NE Atlantic^29^.

For *P. flesus*, earlier spawning migrations in the UK were associated with colder periods^46^, by avoiding lower than average water temperatures at the estuaries where they reside, and thus maximizing their gonadal growth rates before spawning. Delaying of spawning has also been described in freshwater fishes^56^, which can be a consequence of diverting energy from gonadal development to cope with increased metabolic costs of a warmer ocean^5^. *Solea solea* North Sea populations spawn at water temperature higher than 7 °C, and they are expected to spawn earlier in face of a warming ocean, which leads to an earlier arrival of larvae in nurseries, but at the cost of higher mortality due to slow growth early in spring^56^. As such, we hypothesize that warming may benefit populations living presently in colder conditions, such as the North Sea (i.e., faster growth rates, earlier maturation), while those living at lower latitudes (i.e., warmer) such as the Portuguese coast might face additional challenges that can ultimately delay their reproductive and early life phenology. A previous study highlighted that *S. solea* recruitment in the Portuguese coast was higher in “warm” years, while nil recruitment was observed in *P. flesus*, particularly in the southern Iberian coast, most probably because this species is at its southern limit of distribution, and thus, faces additional thermal stress that hampers their ability to cope with increasing water temperatures^57^.

The two main large scale atmospheric circulation patterns in the Northeast Atlantic—North Atlantic Oscillation (NAO) and the East Atlantic pattern (EA), were correlated with a early life phenology of both flounder and sole. Indeed, they are both known to induce variability in oceanic and atmospheric circulation^20^, inter-annual variations in SST, precipitation, wind speed and direction^58,59^, and associated biological responses in marine and estuarine areas^44^. Comparatively to the NAO, the EA is a dipole with a more zonally orientation, with higher influence over southern Europe^20^. However, the NAO and the EA had a contrasting relationship with flounder and sole early life phenology: while increasing NAO was related with earlier hatching, metamorphosis, and settlement in flounder and sole, increasing EA values were synchronous with later hatching and subsequent stages, but only for flounder.

Analyzing the variation of these two climate patterns in the present study, 2011 was the year in which the NAO reached the highest values (2.52) and EA the lowest (− 0.015), which coincided with the earliest start of all life stages in both species: between January–April for *P. flesus*, and between January and May for *S. solea*. From 2010 to 2015, the response to NAO was also similar in both species and life stages. The onset of hatching had a bimodal response, with near zero NAO with later hatches. We also found a negative linear response with metamorphosis and settlement day, indicating that higher NAO is associated with an earlier onset of these stages. Our results match with those recently reported for the European seabass (*Dicentrarchus labrax*)^29^, demonstrating a consistent NAO effect on marine fish species with complex life cycles that involve larval migration from coastal spawning sites into inshore nurseries. At higher latitudes, positive NAO phases during winter are associated to warmer weather^60^, which contribute with more favorable conditions to fish development and growth^61^. However, its direct influence at more southern areas like the Portuguese coast is comparably lower^22^, given its combined effect on ocean temperature^62^, wind and water circulation patterns^63^. Still, the shortest hatch, metamorphosis, and settlement periods for both species occurred in 2010, which was characterized by a strong negative NAO. This situation is consistent with generalized unfavorable weather conditions, cold water and low primary production, promoting small windows of opportunity for fish individual growth and development^64,65^.

The East Atlantic pattern is known as a key driver in inter-annual variations of air and ocean temperature, precipitation, and wind, with ecological consequences at local and regional scales^58^. In our study, the EA pattern had a contrasting trend with the NAO, showing a positive (albeit non-linear) relationship with the onset of hatch, metamorphosis and settlement only on *P. flesus*, indicating that positive EA values influenced their early life phenology. In southern Europe, positive EA values are associated with warmer temperatures, which match the effect of water temperature, at least for flounder. Thus, the influence of the EA pattern can be felt not only on fish physiology and energy allocation, but also on ecosystem productivity and changes in prey-predator interactions. A recent study highlighted that positive EA values led to decreased population growth in deep-sea scorpaenid fishes (*Helicolenus dactylopterus* and *Pontinus kuhlii*), which was mainly due to increased summer temperatures and associated declines in oceanic productivity^21^. Previous studies have also indicated that both the EA and the NAO have the strongest impacts on atmospheric and oceanic processes in the winter^41^, which is the reproductive period of both species^12,14^, and thus, are key drivers for changes in reproductive and early life phenology in marine fishes. However, the EA usually interacts with the NAO, which reflect mainly the latitudinal position and speed of the Atlantic jet stream during the winter^66^. In this case, a composite negative NAO and positive EA lead to increased storminess in the Iberian Peninsula^20^, which based on our results, is linked to a delayed hatching, metamorphosis, and settlement in flounder.

In *P. flesus*, upwelling showed a similar linear positive trend as SST, where more intense upwelling occurred in parallel with a later hatching, metamorphosis, and benthic settlement. This oceanic phenomenon is characterized by the rising of colder and nutrient-rich waters mainly during spring and summer^67^, promoting phytoplankton blooms that increase the zooplankton production^68^, enduring higher food availability for the newly hatched larvae. There has been a constant increase in upwelling in the main upwelling systems worldwide, among which the Iberian coast is included^68^, induced by increasing northerly winds in spring and summer months. This will have a major impact on many biological processes with a seasonal frequency, such as reproduction, early life ontogeny and growth. This is the case of the present work, where more intense upwelling events were related with a later onset of metamorphosis and settlement stages in flounder, which might also cause larvae to be more susceptible to offshore advection, having a direct impact on ocean-estuary connectivity and recruitment.

Connectivity between spawning grounds and estuaries is a key feature in marine fishes, as it shapes population structure, dynamics, and habitat colonization patterns^69^. In this work, we demonstrated that climate and oceanic variability directly impacted on marine flatfishes’ life cycle, particularly during their early life stages and in the transition between the continental shelf and the estuarine nurseries. This was more evident in *P. flesus*, which demonstrated a tighter environmental control, most probably because the Portuguese coast is its southernmost limit of distribution. Further investigations on this topic are encouraged, namely broadening the spatial and temporal coverage, given the links between larval and juveniles stages, recruitment, and fish stocks. Our work contributed to the increasing body of evidence that climate and oceanic variability is directly linked to early ontogenic development and connectivity of marine fishes with complex life cycles.

## Supplementary Information

Supplementary Information.

## Data Availability

The datasets generated during and/or analyzed during the current study are available from the corresponding author on reasonable request.
