# Supplementary material for: Interannual variability in early life phenology is driven by climate and oceanic processes in two NE Atlantic flatfishes
Source: Sci Rep. 2023 Mar 11;13:4057. doi: 10.1038/s41598-023-30384-7 (PMC10008569; doi:10.1038/s41598-023-30384-7)
Supplement: Supplementary file 1 — Supplementary Information. [file 41598_2023_30384_MOESM1_ESM.docx]

**Table S1.** Environmental conditions experienced by *Platichthys flesus* at hatching, metamorphosis, and settlement for each year. Min – minimum values; Max – maximum values.

| **Life stage** | **Year** | **SST (ºC)** | |  | **Chla (mg/L)** | |  | **NAO** | |  | **EA** | |  | **UI** | |
| --- | --- | --- | --- | --- | --- | --- | --- | --- | --- | --- | --- | --- | --- | --- | --- |
|  |  | **Min** | **Max** |  | **Min** | **Max** |  | **Min** | **Max** |  | **Min** | **Max** |  | **Min** | **Max** |
| **Hatch day** | 2010 | 13.91 | 15.53 |  | 0.96 | 11.10 |  | -0.88 | -0.88 |  | 1.23 | 1.23 |  | -448.62 | -448.62 |
|  | 2011 | 13.37 | 17.76 |  | 0.89 | 4.94 |  | -0.88 | 2.48 |  | -1.31 | 0.41 |  | -378.54 | 412.08 |
|  | 2012 | 13.38 | 15.28 |  | 0.48 | 10.94 |  | 0.42 | 1.27 |  | -1.76 | -0.28 |  | 61.60 | 427.61 |
|  | 2013 | 13.40 | 15.95 |  | 1.18 | 5.40 |  | -1.61 | 0.69 |  | -0.24 | 1.31 |  | -778.28 | 1015.23 |
|  | 2014 | 13.37 | 15.54 |  | 1.24 | 18.84 |  | 0.31 | 1.34 |  | 0.52 | 2.23 |  | -823.34 | 51.55 |
|  | 2015 | 12.29 | 14.67 |  | 1.81 | 12.70 |  | 0.73 | 1.79 |  | -0.04 | 1.20 |  | 30.24 | 795.07 |
| **Metamorphosis day** | 2010 | 14.93 | 17.63 |  | 0.58 | 2.41 |  | -0.88 | -0.72 |  | 0.09 | 1.23 |  | -448.62 | -157.38 |
|  | 2011 | 13.78 | 18.97 |  | 1.02 | 4.94 |  | -1.28 | 2.48 |  | -0.77 | 0.41 |  | -168.21 | 661.73 |
|  | 2012 | 13.20 | 17.15 |  | 0.28 | 10.94 |  | -0.91 | 1.27 |  | -1.73 | 0.52 |  | -147.47 | 427.61 |
|  | 2013 | 13.47 | 15.95 |  | 1.18 | 5.74 |  | -1.61 | 0.69 |  | -0.24 | 1.31 |  | -778.28 | 1015.23 |
|  | 2014 | 13.54 | 16.19 |  | 1.24 | 2.80 |  | -0.92 | 0.80 |  | 0.38 | 0.92 |  | -163.01 | 580.99 |
|  | 2015 | 13.37 | 16.19 |  | 1.24 | 18.84 |  | -0.92 | 0.80 |  | 0.71 | 1.20 |  | -163.01 | 580.99 |
| **Settlement day** | 2010 | 15.66 | 17.63 |  | 0.58 | 3.36 |  | -1.49 | -0.72 |  | -1.39 | 0.09 |  | -157.38 | 299.60 |
|  | 2011 | 13.78 | 18.97 |  | 1.05 | 4.94 |  | -1.28 | 2.48 |  | -0.77 | -0.22 |  | -168.21 | 661.73 |
|  | 2012 | 13.20 | 18.68 |  | 0.28 | 10.94 |  | -2.53 | 1.27 |  | -0.64 | 0.52 |  | -147.47 | 327.22 |
|  | 2013 | 13.47 | 15.95 |  | 1.18 | 5.74 |  | -1.61 | 0.69 |  | -0.24 | 1.31 |  | -778.28 | 1015.23 |
|  | 2014 | 13.98 | 16.37 |  | 1.24 | 2.82 |  | -0.92 | 0.31 |  | 0.38 | 0.52 |  | -163.01 | 580.99 |
|  | 2015 | 12.82 | 16.40 |  | 0.54 | 12.70 |  | 0.15 | 1.45 |  | 0.71 | 1.20 |  | 30.24 | 892.59 |

**Table S2**. Environmental conditions experienced by *Solea solea* at hatching, metamorphosis, and settlement for each year. Min – minimum values; Max – maximum values.

| **Life stage** | **Year** | **SST (ºC)** | |  | **Chla (mg/L)** | |  | **NAO** | |  | **EA** | |  | **UI** | |
| --- | --- | --- | --- | --- | --- | --- | --- | --- | --- | --- | --- | --- | --- | --- | --- |
|  |  | **Min** | **Max** |  | **Min** | **Max** |  | **Min** | **Max** |  | **Min** | **Max** |  | **Min** | **Max** |
| **Hatch day** | 2010 | 12.97 | 16.96 |  | 0.96 | 11.10 |  | -1.98 | -0.72 |  | 0.09 | 1.25 |  | -763.93 | -157.38 |
|  | 2011 | 13.78 | 18.97 |  | 1.05 | 3.20 |  | -0.06 | 0.70 |  | -0.77 | 0.41 |  | -168.21 | 412.08 |
|  | 2012 | 13.38 | 15.28 |  | 0.48 | 10.94 |  | 0.42 | 1.27 |  | -0.64 | -0.28 |  | 61.60 | 427.61 |
|  | 2013 | 13.40 | 14.79 |  | 1.18 | 4.61 |  | -1.61 | 0.69 |  | -0.24 | 1.31 |  | -778.28 | 1015.23 |
|  | 2014 | 13.54 | 16.19 |  | 1.24 | 18.84 |  | -0.92 | 0.80 |  | 0.38 | 0.92 |  | -163.01 | 580.99 |
|  | 2015 | 12.29 | 16.40 |  | 0.54 | 12.70 |  | 0.15 | 1.45 |  | 0.71 | 1.20 |  | 30.24 | 892.59 |
| **Metamorphosis day** | 2010 | 14.23 | 17.63 |  | 0.58 | 11.10 |  | -1.49 | -0.72 |  | -1.39 | 1.23 |  | -448.62 | 299.60 |
|  | 2011 | 13.78 | 18.87 |  | 1.02 | 4.94 |  | -1.28 | 2.48 |  | -0.77 | -0.22 |  | -9.50 | 661.73 |
|  | 2012 | 13.20 | 15.28 |  | 0.28 | 10.94 |  | -0.91 | 1.27 |  | -0.64 | 0.52 |  | -147.47 | 327.22 |
|  | 2013 | 13.47 | 15.95 |  | 1.18 | 5.40 |  | -1.61 | 0.69 |  | -0.24 | 1.31 |  | -778.28 | 1015.23 |
|  | 2014 | 13.98 | 19.22 |  | 0.28 | 2.82 |  | -2.53 | 0.31 |  | -1.03 | 0.52 |  | -163.01 | 580.99 |
|  | 2015 | 12.82 | 16.40 |  | 0.76 | 22.21 |  | -0.07 | 1.45 |  | 0.71 | 1.20 |  | 30.24 | 892.59 |
| **Settlement day** | 2010 | 14.93 | 17.63 |  | 0.58 | 11.10 |  | -1.49 | -0.72 |  | -1.39 | 1.23 |  | -448.62 | 299.60 |
|  | 2011 | 13.85 | 18.51 |  | 1.02 | 5.59 |  | -1.51 | 2.48 |  | -0.77 | -0.22 |  | -9.50 | 1148.75 |
|  | 2012 | 13.20 | 17.15 |  | 0.28 | 10.94 |  | -0.91 | 1.27 |  | -0.24 | 1.31 |  | -147.47 | 327.22 |
|  | 2013 | 13.82 | 15.95 |  | 1.18 | 5.40 |  | 0.57 | 0.69 |  | 0.14 | 1.31 |  | 471.19 | 1015.23 |
|  | 2014 | 14.98 | 19.22 |  | 0.28 | 2.82 |  | -0.97 | 0.31 |  | -1.03 | 0.52 |  | -163.01 | 580.99 |
|  | 2015 | 14.27 | 17.05 |  | 0.54 | 22.21 |  | -0.07 | 1.45 |  | 0.71 | 1.20 |  | 30.24 | 892.59 |

**Table S3**. Results of the Wilcoxon pairwise test for multiple comparisons (with Bonferroni correction) for hatch, metamorphosis, and settlement days for *Platichthys flesus* from 2010 to 2015. Significant terms are highlighted with *.

|  | 2010 | 2011 | 2012 | 2013 | 2014 |
| --- | --- | --- | --- | --- | --- |
| *Hatch day* |  |  |  |  |  |
| 2011 | 0.55024 |  |  |  |  |
| 2012 | 1.00000 | 1.00000 |  |  |  |
| 2013 | 0.87167 | 0.00025* | 0.00111* |  |  |
| 2014 | 1.00000 | 0.02829* | 0.19227 | 1.00000 |  |
| 2015 | 0.42440 | 1.00000 | 1.00000 | 5.7e^-05^* | 0.00874* |
| *Metamorphosis day* |  |  |  |  |  |
| 2011 | 0.69289 |  |  |  |  |
| 2012 | 1.00000 | 1.00000 |  |  |  |
| 2013 | 1.00000 | 0.00058* | 0.00172* |  |  |
| 2014 | 1.00000 | 0.02411* | 0.07020 | 1.00000 |  |
| 2015 | 0.48419 | 1.00000 | 1.00000 | 0.00018* | 0.00440* |
| *Settlement day* |  |  |  |  |  |
| 2011 | 0.40424 |  |  |  |  |
| 2012 | 1.00000 | 1.00000 |  |  |  |
| 2013 | 1.00000 | 0.00041* | 0.00122* |  |  |
| 2014 | 1.00000 | 0.01278* | 0.05104 | 1.00000 |  |
| 2015 | 0.25106 | 1.00000 | 1.00000 | 7.2e^-05^* | 0.00193* |

**Table S4**. Results of the Wilcoxon pairwise test for multiple comparisons (with Bonferroni correction) for hatch, metamorphosis, and settlement days for *Solea solea* from 2010 to 2015. Significant terms are highlighted with *.

|  | 2010 | 2011 | 2012 | 2013 | 2014 |
| --- | --- | --- | --- | --- | --- |
| *Hatch day* |  |  |  |  |  |
| 2011 | 1.00000 |  |  |  |  |
| 2012 | 0.81710 | 1.00000 |  |  |  |
| 2013 | 1.00000 | 1.00000 | 1.00000 |  |  |
| 2014 | 0.0037* | 1.00000 | 0.00450* | 0.00042* |  |
| 2015 | 9.8e^-06^* | 1.00000 | 1.1e^-05^* | 2.1e^-05^* | 1.00000 |
| *Metamorphosis day* |  |  |  |  |  |
| 2011 | 1.00000 |  |  |  |  |
| 2012 | 1.00000 | 1.00000 |  |  |  |
| 2013 | 1.00000 | 1.00000 | 1.00000 |  |  |
| 2014 | 0.0012* | 1.00000 | 0.0381* | 0.0018* |  |
| 2015 | 8.6e^-06^* | 1.00000 | 5.1e^-06^* | 1.6e^-05^* | 1.00000 |
| *Settlement day* |  |  |  |  |  |
| 2011 | 1.00000 |  |  |  |  |
| 2012 | 1.00000 | 1.00000 |  |  |  |
| 2013 | 1.00000 | 1.00000 | 1.00000 |  |  |
| 2014 | 0.00083* | 1.00000 | 0.03365* | 0.00047* |  |
| 2015 | 1.3e^-05^* | 1.00000 | 1.5e^-05^* | 8.0e^-06^* | 1.00000 |
